# ECMO-Supported Ablation and Percutaneous Repair of Severe Valvulopathy: A Winning Combination in a Complex Clinical Case

**DOI:** 10.3390/jcdd8120188

**Published:** 2021-12-15

**Authors:** Federica Troisi, Katya Lucarelli, Vito Casamassima, Tommaso Langialonga, Rosa Caruso, Nicola Duni, Federico Quadrini, Antonio Di Monaco, Nicola Vitulano, Massimo Grimaldi

**Affiliations:** 1 Cardiology Department, Regional General Hospital “F. Miulli”, Acquaviva delle Fonti, 70021 Bari, Italy; k.lucarelli@miulli.it (K.L.); V.casamassima@miulli.it (V.C.); t.langialonga@miulli.it (T.L.); r.caruso@miulli.it (R.C.); n.duni@miulli.it (N.D.); f.quadrini@miulli.it (F.Q.); a.dimonaco@miulli.it (A.D.M.); n.vitulano@miulli.it (N.V.); m.grimaldi@miulli.it (M.G.); 2 Department of Clinical and Experimental Medicine, University of Foggia, 71121 Foggia, Italy

**Keywords:** acute heart failure, electrical storm, severe mitral insufficiency, winning combination therapies, teamwork

## Abstract

In this case report, we describe a complex case of a 67-year-old patient who was suffering from acute heart failure with electrical storm. Clinical case management was based on an integrated approach comprising two different procedures that were complementary and synergistic, and that allowed the patient to reach acute stabilization and to demonstrate mid-term clinical improvement. Complex clinical settings, such as electrical and hemodynamic instability, require complex solutions. The use of an integrated approach that allows physiopathological mechanisms to work together may be beneficial for these patients.

## 1. Introduction

Acute heart failure can be a difficult condition to manage, since it can be the consequence of various decompensation mechanisms that have been in place for a long period of time. In these situations, it is known that it may be necessary to act pharmacologically on several fronts in order to try to interrupt the negative and vicious cycles that have been triggered by acute heart failure. The same integrated approach may also be necessary in interventional therapeutic strategies.

## 2. Case Report

A sixty-seven-year-old man was admitted to our hospital while suffering from electrical instability of the heart, resistant to the antiarrhythmic drugs that are often used to treat a patient with infarct heart disease (metoprolol, amiodarone, magnesium sulfate, potassium chloride, and xylocaine). The patient had diabetes, suffered from chronic hypertension and bronchitis, was affected by ischemic heart disease, had already been subjected to surgical and percutaneous myocardial revascularization, and had a clinical history of acute cardiac decompensations, despite having received optimized medical therapy for heart failure.

**Figure 1:**
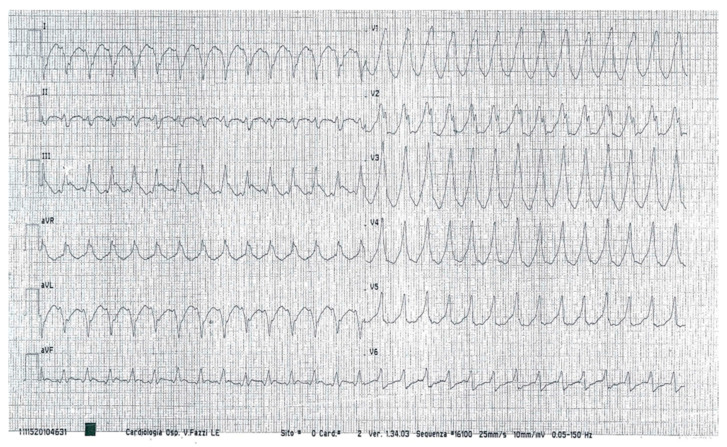
The twelve-lead ECG recorded a monomorphic and sustained ventricular tachycardia, originating from the left ventricle.

The patient was taken to another hospital while suffering from acute chest pain, which was complicated by cardiac arrest (ventricular fibrillation). He immediately underwent a coronary angiography (negative for new coronary stenoses), and he was implanted with a bicameral implantable cardioverter defibrillator (ICD) a few days later. During a subsequent hospitalization, he had unfortunately received several shocks from the ICD as a result of sustained ventricular tachycardia (Figure 1), despite receiving antiarrhythmic drug therapy (metoprolol 100 mg die, amiodarone 200 mg die, magnesium sulfate, and potassium chloride via continuous i.v. infusion).

**Figure 2:**
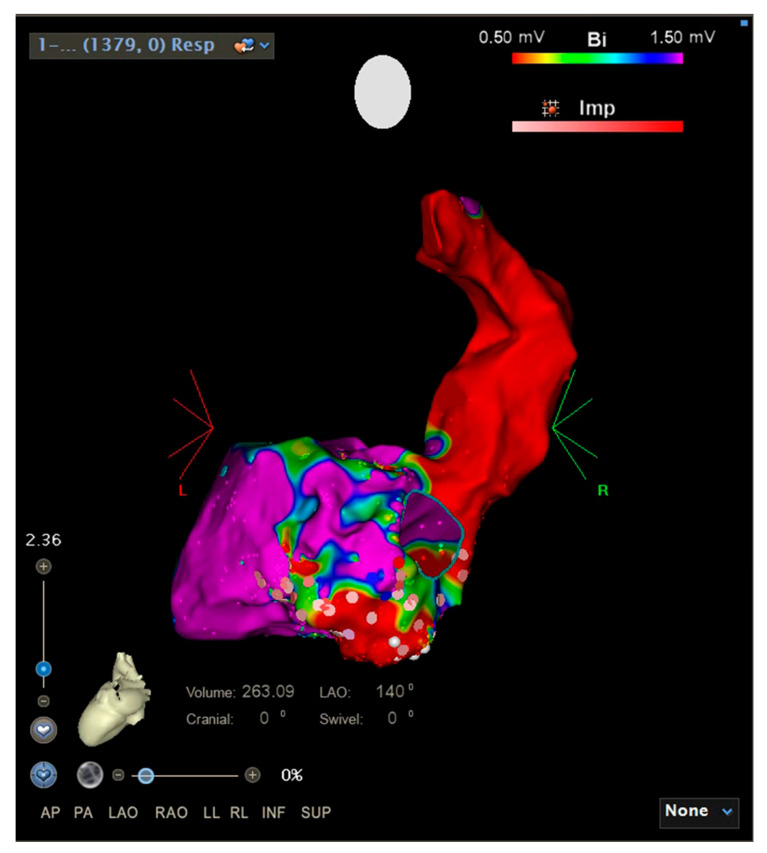
Electroanatomic map of the left ventricle at the end of ablation: low voltage areas (red in color) correspond to the infarct scar, pink points are the ablation points.

At admission to our hospital, his clinical condition was critical: he could not breathe without ventilatory support (NPPV—Non-Invasive Positive Pressure Ventilation) and he demonstrated continuous electrical status instability. A transthoracic echocardiogram was performed. Despite severe mitral regurgitation, the left ventricular ejection fraction was reduced (estimated at 40%). We decided to perform a ventricular tachycardia ablation [1], using cardiopulmonary support by means of extracorporeal membrane oxygenation (ECMO), because of the hemodynamic instability of the patient during arrhythmia [2]. The use of the ECMO system secured hemodynamic stability throughout the procedure; therefore, the ECMO system facilitated the creation of an accurate voltage mapping of the patient’s heart and the ablation of the unstable ventricular arrhythmias. Low-voltage areas were identified in the lateral basal, inferolateral basal, and posterior zone of the left ventricle of our patient, and radiofrequency erogations were applied in these areas (Figure 2).

**Figure 3:**
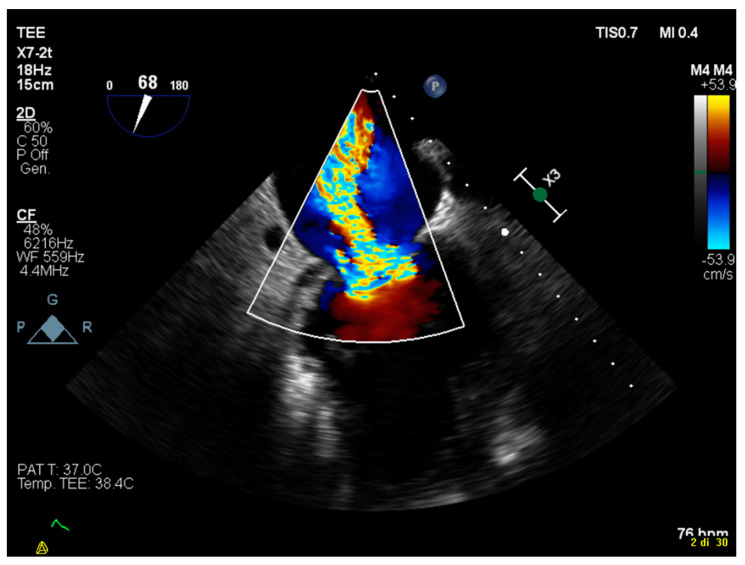
An intercommissural view on the transesophageal echocardiogram highlights severe mitral insufficiency with prevalent mitral regurgitation jet from the A2-P2 mitral valve scallops.

During the ablation, the patient began to demonstrate ventricular tachycardia with a morphology that was compatible with that of clinical arrhythmia, but the patient remained hemodynamically stable because of the ECMO system. At the end of the procedure, an inducibility study was conducted, the results of which were negative for ventricular arrhythmia. During the following days, the patient became electrically stable. While the patient was in a favorable clinical condition, we conducted a transesophageal echocardiogram, which indicated a severe mitral insufficiency with a prevalent mitral regurgitation jet that appeared to have originated from the A2–P2 mitral valve scallops (Figure 3). Because of this, the patient was eligible for percutaneous correction of this valvular defect.

**Figure 4:**
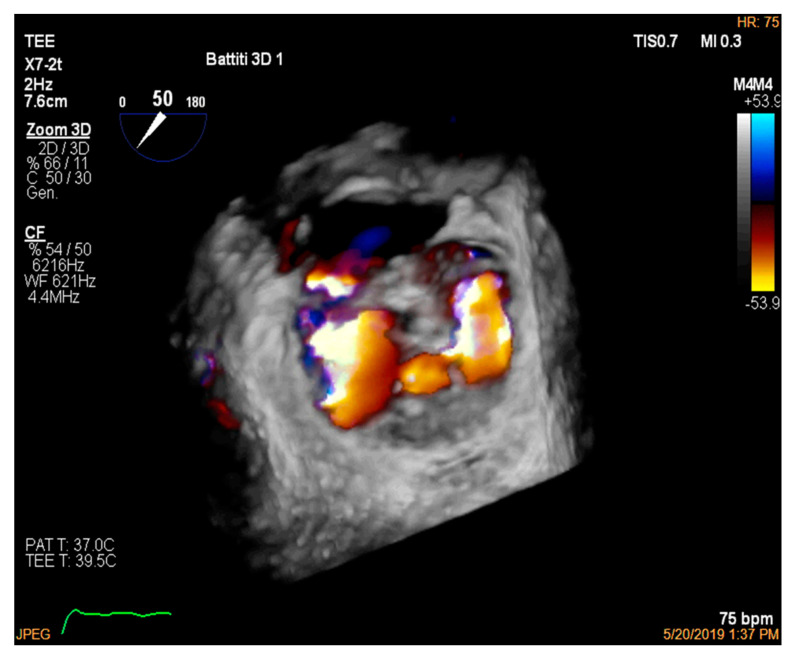
A live zoom 3D view at the end of the percutaneous correction procedure highlights a single centrally positioned clip with residual mild mitral insufficiency.

After the patient had been clinically and electrically stable for one week, the patient was discharged and prescribed optimized heart failure therapy (metoprolol 200 mg die, furosemide 350 mg die, ramipril 5 mg die, potassium canrenoate 100 mg die). Two weeks later, he returned to our hospital, and we performed a percutaneous mitral regurgitation correction procedure with a double MitraClip placement, which was performed using a transesophageal echocardiographic guide [3,4,5]. The procedure achieved a mitral insufficiency reduction, with a decrease from grade IV to grade I being observed, and a final transvalvular mean mitral gradient (PGMm) of 4 mmHg (Figure 4).

The patient attended follow-up appointments at three months, six months, and one year after the last hospital procedure. The ICD had recorded neither device interventions nor sustained arrhythmias. A transthoracic echocardiogram showed very good results: residual mitral insufficiency was mild (with PGMm 4 mmHg), and the left ventricular ejection fraction was estimated to be 45%. The patient was hemodynamically stable. The patient stated that he was no longer using home oxygen therapy, which he had reported using in the past (before hospitalization for electrical instability). Therefore, there was a noticeable increase in the patient’s quality of life: his Minnesota Living with Heart Failure Questionnaire (MLHFQ) score decreased from 84 (measured during hospitalization before percutaneous correction of the valvular insufficiency was performed) to 48 (measured as early as the three-month follow-up and remained stable at subsequent follow-up visits).

## 3. Discussion

We report the case of a patient who was suffering from severe cardiac failure and who was affected by electrical and hemodynamic instability with two different and complementary percutaneous procedures that resulted in dramatic clinical improvement in a short period of time. In particular, the first procedure, ECMO-supported ablation for ventricular arrhythmia, allowed the patient to achieve acute stabilization [6]; the second intervention, percutaneous valvular defect repair, led to an improvement in the patient’s mid-term prognosis, preventing new heart failure decompensation. Patients suffering from electrical instability are very complex, especially those with arrhythmias and concomitant structural heart disease. The reason for this is that heart failure triggers arrhythmias, but arrhythmias also trigger heart failure. This clinical setting probably requires an integrated approach, such as the one presented here, that is both electric and hemodynamic.
